# Mineral Admixture Governs the Synergy of Polymer and Fibers in Ultra-Low Temperature Concrete

**DOI:** 10.3390/ma19081541

**Published:** 2026-04-12

**Authors:** Yao Li, Yonggang Deng

**Affiliations:** School of Architecture and Civil Engineering, Liuzhou Institute of Technology, Liuzhou 545616, China; 13998802511@163.com

**Keywords:** cryogenic durability, redispersible polymer powder, silica fume, fiber reinforcement, matrix compatibility

## Abstract

**Highlights:**

- Polymer fiber synergy in cryogenic concrete is mineral admixture-dependent.
- Silica fume enables best performance via dense matrix, continuous polymer film, and fiber bridging.

**Abstract:**

The development of all-concrete liquefied natural gas (LNG) storage tanks is hindered by the susceptibility of conventional concrete to ultra-low temperature (ULT) cycling down to −70 °C. While redispersible polymer powder (RPP) and polypropylene (PP) fibers individually enhance performance, their combined effect in various mineral admixture systems remains unclear. This study investigates the synergy and selective compatibility in hybrid-modified concrete containing fly ash (FA), silica fume (SF), or slag (SG). Comprehensive assessments after 50 ULT cycles reveal that the efficacy of hybrid modification is intrinsically governed by the mineral admixture. Among all systems, the silica fume-based hybrid system (EPSF) exhibits the highest residual compressive strength (57.5 MPa), the lowest strength loss (6.7%), and the most balanced durability. Microstructural analysis reveals that this synergy arises from a dense matrix, continuous polymer network, and effective fiber bridging—achieved only when the mineral admixture enables uniform RPP distribution. In contrast, the FA system exhibits a strength–durability trade-off, with RPP localized at interfaces, while the SG system shows a polymer-activated hydration mechanism. Microstructural and nano-mechanical analyses confirm that RPP acts as a pore filler in cement, an interfacial modifier in FA, a cohesive network former in SF, and a hydration activator in SG. This work establishes that superior ULT resilience requires not merely additive modifications but a matrix-enabled synergy, providing a scientific basis for the rational design of cryogenic concrete.

## 1. Introduction

The development of robust liquefied natural gas (LNG) storage infrastructure is a critical enabler of the global transition to cleaner energy [1]. All-concrete LNG (ACLNG) storage tanks offer a compelling alternative to traditional, high-cost nickel-steel systems, providing significant advantages in construction efficiency and cost reduction [2,3]. However, realizing this potential critically depends on the concrete’s ability to withstand an exceptionally aggressive service environment: sustained ultra-low temperatures (ULT) approaching −165 °C, coupled with severe thermal cycling. The most damaging freeze–thaw stresses often occur at intermediate subzero temperatures, particularly around −70 °C, where pore ice formation and expansion induce significant microstructural damage [4,5].

Extensive research over recent decades has established that concrete subjected to cryogenic conditions exhibits complex and often contradictory behavior. On one hand, compressive strength typically increases with decreasing temperature due to the freezing of pore water, densifying the matrix and enhancing its ice-based load-bearing capacity [6,7]. Cheng et al. [2] recently demonstrated that a high-strength cryogenic concrete (HCC) achieves maximum compressive strength enhancement at −45 °C, which correlates with the freezing of gel pore water in the −35 °C to −45 °C range. Conversely, tensile strength—a more damage-sensitive parameter—typically peaks at moderate subzero temperatures and then declines at deeper cryogenic levels, reflecting the onset of microcracking from ice crystallization pressure and thermal incompatibility between constituents [8]. This strength–damage duality underscores the inherent vulnerability of cementitious materials under ULT exposure.

To elucidate the origins of this vulnerability, the degradation mechanisms under cryogenic freeze–thaw cycling have attracted increasing scholarly attention. Studies have shown that repeated freezing and thawing at ultra-low temperatures progressively deteriorates pore structure, transforms gel pores into larger capillary pores, and ultimately initiates and propagates microcracks [9,10]. Using acoustic emission techniques, Kogbara et al. [11] correlated thermal deformation with microcracking during cryogenic cooling, revealing that the most intense damage occurs in the −20 °C to −70 °C range. Nuclear magnetic resonance (NMR) investigations have further confirmed that freeze–thaw cycling increases total porosity and shifts pore size distributions towards larger, more harmful pores [12]. These microscopic alterations manifest macroscopically as strength loss, reduced elastic modulus, and increased permeability [13,14]. The literature on cryogenic concrete has developed along three distinct lines of inquiry. European researchers—notably Rostasy et al. [9] and Miura [4]—laid the groundwork for understanding pore ice formation and its mechanical implications. North American research, as exemplified by Kogbara et al. [5,11], has concentrated on nondestructive evaluation and mixture design for direct LNG containment. Asian researchers—particularly Jiang et al. [12], and more recently Cheng et al. [2] and Li et al. [15]—have provided detailed microstructural characterizations and predictive models. Despite these regional contributions, no study has systematically investigated how polymer, fiber, and mineral admixture function together as an integrated system under cryogenic conditions.

To mitigate these deleterious effects, researchers have explored various modification strategies, each targeting a specific aspect of concrete’s vulnerability. Mineral admixtures (MAs) such as silica fume (SF), fly ash (FA), and ground granulated blast-furnace slag (SG) are widely employed to refine pore structure and enhance durability through pozzolanic reactions and microfilling effects [16,17]. Polypropylene (PP) fibers provide crack-bridging capability, thereby improving toughness and controlling crack propagation under thermal stress [18,19]. More recently, redispersible polymer powders (RPPs) have been introduced to modify the cementitious matrix at the micro-to-nanoscale, forming flexible films that enhance cohesion, reduce permeability, and improve stress redistribution [20,21]. Each approach has demonstrated measurable benefits under controlled conditions.

While the efficacy of each individual approach has been well established, the literature has largely examined them in isolation. This has created a critical blind spot concerning their combined interactions within a single system. This knowledge gap is especially critical in the context of ULT cycling. Under these conditions, the simultaneous requirements for matrix densification, crack control, and flexibility demand a level of synergy that cannot be achieved through simple additive effects. Although Cheng et al. [2] established the importance of matrix densification for cryogenic concrete performance, subsequent studies have expanded this understanding along complementary lines. da Cunha-Oliveira et al. [22] showed that low-temperature treated sewage sludge can act as an effective filler in ultra-high-performance concrete, implying that unconventional mineral admixtures may confer cryogenic benefits. Li et al. [15] systematically characterized the stress–strain relationship of ultra-high-performance concrete containing coarse aggregate under ultra-low temperatures, revealing that aggregate type significantly influences the mechanical response. Rong et al. [23] investigated reactive powder concrete under cryogenic compression and identified a transition in failure mode as temperature decreased. Cai et al. [24] proposed a method for predicting the macroscopic mechanical properties of concrete at ultra-low temperatures based on pore structure evolution. Despite these advances, the specific role of interactions among polymer, fiber, and mineral admixture in imparting resilience to ultra-low temperatures remains unexplored. Moreover, although Zeng et al. [25] examined fiber-reinforced polymer composites for concrete columns and Feng et al. [26] studied FRP-strengthened beams in coastal environments, neither investigated the ternary hybrid system (mineral admixture + polymer + fiber) under cryogenic thermal cycling.

Prior research has primarily focused on the individual benefits of mineral admixtures, fibers, or polymers under cryogenic conditions. However, a more complex and practically relevant question remains: does the combined incorporation of redispersible polymer powder (RPP) and polypropylene (PP) fibers universally enhance performance across all matrices, or is their hybrid modification selectively more effective with specific types of mineral admixtures. Critically, the existing literature offers no guidance on whether the mechanisms of RPP (e.g., film formation) and PP fibers (e.g., crack bridging) are complementary or competitive within matrices of varying density and reactivity, such as fly ash versus silica fume. This knowledge gap hinders the rational design of high-performance cryogenic concrete because the critical role of the matrix—the very platform that hosts these modifiers—remains unexplored.

Therefore, this study aims to address this gap by shifting the research focus from the effects of individual additives to the concept of matrix-enabled synergy. A comprehensive investigation is presented into the synergy and selectivity of concrete modified with a hybrid system of RPP and PP fibers, in combination with various mineral admixtures (FA, SF, SG), under ULT conditions. Specifically, the following aspects are evaluated: (1) the evolution of mechanical properties after ULT cycling, distinguishing between compressive and the more damage-sensitive splitting tensile strength; (2) a suite of durability indicators, including frost resistance, carbonation depth, and early age cracking behavior; (3) microstructural evolution using SEM to correlate macroscopic properties with matrix morphology and interface characteristics; and (4) hydration kinetics via isothermal calorimetry to provide a thermodynamic and kinetic perspective on the observed phenomena. By establishing a fundamental structure–property–performance relationship for these complex, multicomponent systems under extreme thermal conditions, this work aims to provide a scientific foundation for tailoring material compositions, thereby enabling predictable and superior durability in critical cryogenic applications.

## 2. Materials and Methods

### 2.1. Raw Materials

The primary binder used in this study was ordinary Portland cement (42.5 grade, density 3.15 g/cm^3^, Shenyang, China), which was supplemented with three supplementary cementitious materials: Class F fly ash (FA, density 2.35 g/cm^3^, Qingdao, China), silica fume (SF, density 2.20 g/cm^3^, Hengshui, China), and ground granulated blast-furnace slag (SG, Grade S95, density 2.90 g/cm^3^, Shenyang, China). Quartz sand (fineness modulus 2.6, density 2.65 g/cm^3^, Shenyang, China) served as the fine aggregate, and crushed stone (5–20 mm, density 2.70 g/cm^3^, Shenyang, China) served as the coarse aggregate. A polycarboxylate-based superplasticizer (SP, Shenyang, China), with a water reduction rate of 20–25%, was incorporated to ensure adequate workability. The primary modifying agents were polypropylene (PP, Shandong, China) fibers (length: 12 mm; density: 0.91 g/cm^3^; tensile strength: 450 MPa; elastic modulus: 4.5 GPa; melting point: 165 °C) and a redispersible polymer powder (RPP, density 0.50 g/cm^3^, Shandong, China) based on a vinyl acetate–ethylene (VAc/E) copolymer. Municipal tap water served as the mixing water. The chemical compositions of the cementitious materials are presented in Table 1. Table 2 summarizes the key physical and technical parameters of the RPP.

### 2.2. Mixture Design

All concrete mixtures were prepared with a constant water-to-binder (w/b) ratio of 0.35. The control mixture, designated as JZ (from the Chinese “Ji Zhun”, meaning reference or standard), contained cement as the sole binder. Three primary systems were prepared by substituting 20% fly ash (FA), 6% silica fume (SF), or 20% slag (SG) for an equivalent mass of cement, yielding mixes FA, SF, and SG, respectively. Polypropylene (PP) fibers were then incorporated at a constant volume fraction of 0.4% into these mineral admixture systems, producing mixes PFA, PSF, and PSG. Finally, 6% redispersible polymer powder (RPP) by binder mass was incorporated into the fiber-reinforced mixes to investigate the synergistic effect, yielding mixes EPFA, EPSF, and EPSG. Table 3 lists the detailed mix proportions.

The replacement levels for the mineral admixtures and RPP were established through preliminary mechanical and workability tests. For the mineral admixtures, the selected dosages (20% FA, 6% SF, 20% SG) were identified as optimal for balancing compressive strength development with the workability of the fresh mixture. A 6% RPP dosage was adopted, as this was found to promote effective polymer film formation in preliminary trials. Higher dosages were observed to reduce compressive strength, an effect likely attributable to excessive polymer film interference with the cement hydration network. Consequently, these specific proportions were selected to ensure that each modifier contributes effectively within the hybrid system.

The workability of all fresh mixtures was evaluated through slump tests, conducted in accordance with Ref [27]. All mixtures exhibited satisfactory workability for casting, with slump values ranging from 180 mm to 220 mm. The observed increase in workability for the fly ash mixture (FA: 220 mm) is attributed to the spherical morphology of its particles. Conversely, the inclusion of silica fume (SF: 185 mm) reduced workability, a consequence of its high specific surface area. The incorporation of RPP was also found to marginally improve the workability of the hybrid systems. These results confirm that adequate workability was achieved across all mixtures with the superplasticizer dosage fixed at 5.07 kg/m^3^.

### 2.3. Experimental Program

#### 2.3.1. Specimens Preparation

All concrete mixtures were prepared using a laboratory mixer. Cubic specimens measuring 100 mm × 100 mm × 100 mm were cast in triple-gang steel molds according to the mix proportions detailed in Table 3. The specimens were demolded after 24 h and then cured under standard conditions (20 ± 2 °C, ≥95% RH) until 28 days.

#### 2.3.2. Mechanical Properties Testing

To evaluate the mechanical performance of concrete before and after ULT cycling, compressive strength and splitting tensile strength of the concrete mixtures were determined at 28 days according to Ref [28]. For compressive strength testing, cubic specimens (100 mm) were loaded to failure using a universal testing machine. Compressive strength (*f*_c_) was calculated as the peak load (*F*) divided by the bearing area (*A*), according to Equation (1). Splitting tensile strength *(f*_t_) was determined by applying a diametrical line load to a cubic specimen. The strength was computed from the failure load and splitting area using Equation (2). Each mix was tested in triplicate, and the mean strength values are reported.

(1) $$f_{c}=\frac{F}{A}$$

where *F* is the failure load (N) and A is the loading area (=100 cm^2^).

(2) $$f_{t}=\frac{2F_{t}}{\pi A_{t}}=0.637\frac{F_{t}}{A_{t}}$$

where *F*_t_ is the failure load (N) and *A*_t_ is the area of the splitting surface (=100 cm^2^).

#### 2.3.3. Freeze–Thaw Resistance Test

**Figure 1:**
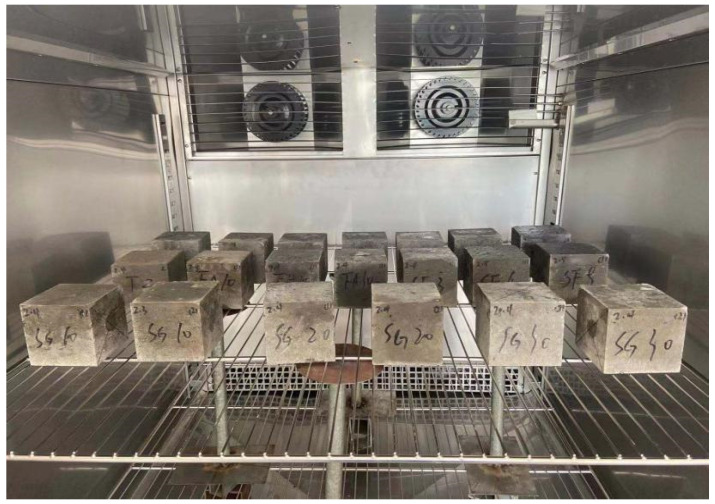
High-low temperature environmental chamber with concrete specimens placed inside for ultra-low temperature freeze–thaw cycling.

To assess the frost resistance of concrete under ultra-low temperature conditions, a freeze–thaw cycling test was conducted. After 28 d of standard curing, cubic specimens were water-saturated by 24 h immersion and then placed in a high–low temperature environmental chamber (Figure 1).

The specimens were subjected to 50 rapid freeze–thaw cycles in a high-low temperature environmental chamber, with temperatures cycling between +20 °C and −70 °C. The cooling and heating rates were maintained at approximately 0.5 °C/min to minimize thermal shock and ensure a uniform temperature distribution within the specimens. Each complete cycle lasted approximately 6 h and consisted of three phases: (1) cooling from +20 °C to −70 °C (approx. 3 h); (2) a 120 min (2 h) holding phase at −70 °C; and (3) heating from −70 °C back to +20 °C (approx. 1 h). To verify that the target temperature was reached and maintained as specified, the core temperature of control specimens was monitored throughout the cycling process. This was achieved using thermocouples embedded at the center of the specimens. These thermocouples were connected to a data acquisition system, which recorded temperatures at 5 min intervals. After cycling, mass loss, compressive strength loss, and relative dynamic elastic modulus were measured to assess frost damage. Mass loss rate for each specimen (Δ*W*_ni_) and the group average (Δ*W*_n_) were calculated using Equations (3) and (4), respectively. Compressive strength loss rate (Δ*f*_c_) was determined by comparing the strength before (f_c0_) and after (*f*c_n_) cycling, according to Equation (5). The relative dynamic elastic modulus (*E*_n_)—an indicator of internal damage—was calculated from the change in fundamental transverse frequency measured using a dynamic modulus tester, according to Equation (6).

(3) $$\Delta w_{n_{i}}=\frac{w_{0i}-w_{ni}}{w_{0i}}\;\times \;100$$

(4) $$\Delta w_{n}=\frac{\sum_{i=1}^{3}\Delta w_{n_{i}}}{3}\;\times \;100$$

where *W*_0i_ and *W*_ni_ are the masses (g) of the *i*th specimen before and after *n* cycles.

(5) $$\Delta f_{c}=\frac{f_{c0}-f_{\mathrm{cn}}}{f_{c0}}\;\times \;100\%$$

(6) $$E_{n}=\frac{f_{n}^{\;2}}{f_{0}^{\;2}}\;\times \;100\%$$

where *f*_0_ and *f*_n_ represent the initial and post-cycling frequencies.

#### 2.3.4. Carbonation Depth Test

**Figure 2:**
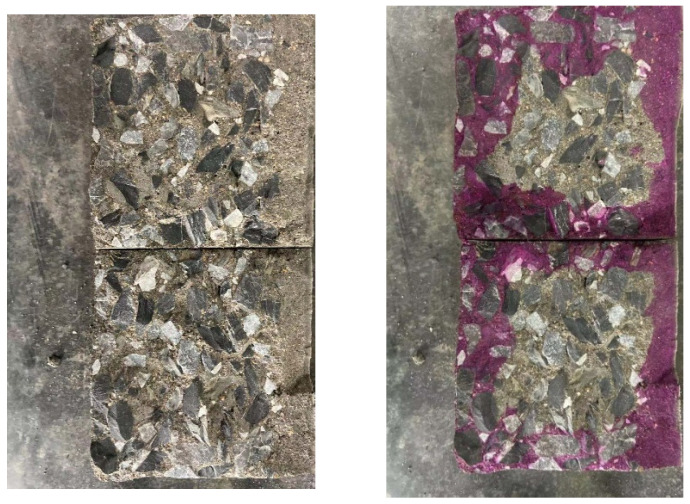
Carbonation of concrete specimen.

To evaluate the long-term durability of concrete under coupled environmental attack, carbonation depth was measured after natural carbonation exposure. Specimens (100 mm cubes) were exposed to natural carbonation for 28 d and 150 d. After exposure, the specimens were split open. The freshly exposed cross-section was cleaned and sprayed with a 1% phenolphthalein–ethanol solution. The colorless region indicated the carbonation depth. Carbonation depth was measured at 36 premarked points (9 points per edge) using vernier calipers, and the average value was recorded (Figure 2).

#### 2.3.5. Early Age Cracking Test

To investigate the plastic shrinkage cracking propensity of fresh concrete, early age plastic shrinkage cracking propensity was evaluated following Ref [29]. A slab specimen (800 mm × 600 mm × 100 mm) with preformed crack inducers was used. After casting and finishing, the slab was immediately exposed to fan-induced wind at a controlled velocity of 5 ± 0.5 m/s parallel to its surface. Crack development was monitored at 24, 48, and 72 h after casting. Crack width was measured with a crack width microscope.

#### 2.3.6. Hydration Heat Analysis

To understand the influence of RPP and mineral admixtures on hydration kinetics, heat flow and cumulative heat release were measured using isothermal calorimetry. Hydration kinetics of cementitious pastes (with binder compositions matching those of the concrete mixes) were measured using an 8-channel TAM Air isothermal calorimeter (TA Instruments, New Castle, DE, USA) at 25 °C for 144 h. Immediately after water mixing, the paste sample was sealed in an ampoule and placed in the calorimeter to monitor heat flow and cumulative heat release.

#### 2.3.7. Microstructural Analysis

To correlate macroscopic properties with matrix morphology and interface characteristics, microstructural analysis was conducted with a Hitachi S-3400N scanning electron microscope (SEM, Hitachi High-Technologies Corporation, Tokyo, Japan). Small fragments (approximately 10 mm × 10 mm) were extracted from the cement paste matrix of selected specimens. To arrest hydration, the fragments were immediately immersed in ethanol after extraction. Prior to imaging, the samples were oven-dried at 50 °C for 1 h and sputter-coated with gold to ensure conductivity.

#### 2.3.8. Nanoindentation

To investigate the nano-mechanical properties of the cementitious matrix, The local elastic modulus of individual phases within the cementitious matrix was measured using nanoindentation. The tested specimens were 30 mm in diameter and 8 mm in height. A 10 × 10 indentation grid was used, with a spacing of 6 µm between indentations. A Poisson’s ratio of 0.25 was assumed for the indentation analysis, which is a commonly adopted value for cement-based materials [30]. A linear loading protocol was applied, with loading and unloading rates of 200 μN/s. The load was increased to a maximum of 2000 μN and held for 30 s.

## 3. Results and Discussion

### 3.1. Mechanical Properties Under Ultra-Low Temperature Freeze–Thaw Cycling

**Figure 3:**
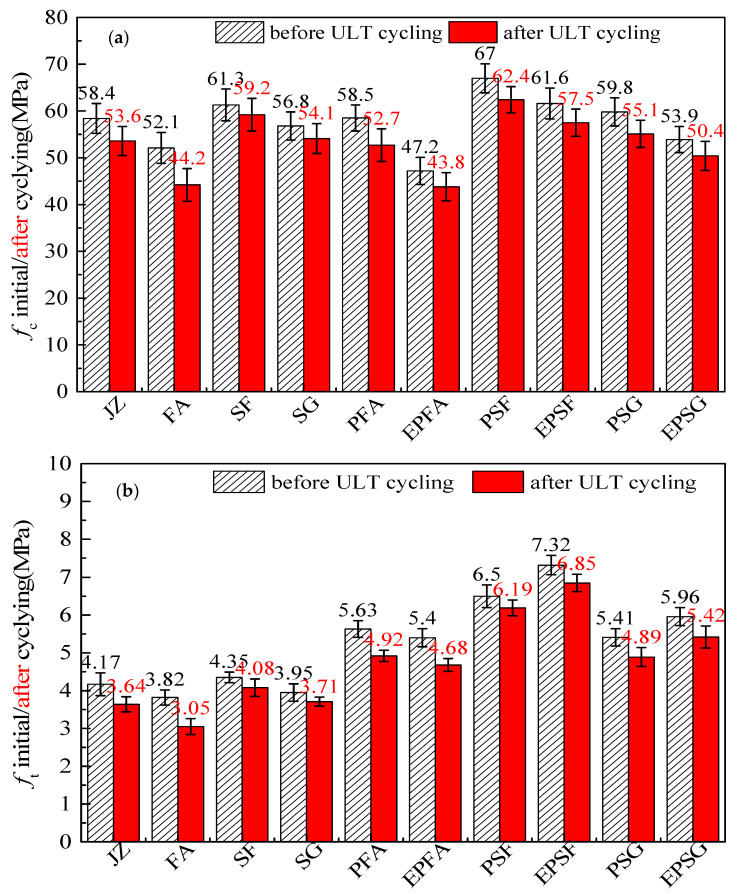
Mechanical properties before and after ULT cycling: (**a**) compressive strength; (**b**) splitting tensile strength. (50 cycles, −70 °C to +20 °C).

The mechanical response of concrete to ultra-low-temperature freeze–thaw cycling reveals a critical finding. The hybrid modification with redispersible polymer powder (RPP) and polypropylene (PP) fibers exhibits selective compatibility fundamentally governed by the mineral admixture type. As shown in Figure 3, the silica fume (SF) system exemplifies a synergistic paradigm: the EPSF mix (SF with both RPP and fiber) achieves the most balanced performance. After 50 cycles, it exhibits the highest residual compressive strength (57.5 MPa), the lowest strength loss (6.7%), and superior splitting tensile strength (7.32 MPa initial, 6.85 MPa after cycling). In contrast, the fly ash (FA) system exhibits a clear strength–durability trade-off. The EPFA mix shows a 19.4% reduction in initial compressive strength relative to its fiber-only counterpart (PFA), although it retains strength marginally better after cycling. The slag (SG) system displays an intermediate response. Across all systems, PP fibers consistently enhance splitting tensile strength, with increases of 31–49% before cycling—a benefit that persists after ULT exposure. The inherent superiority of the SF matrix—evidenced by its minimal strength loss even without modification (3.5%), provides an optimal platform for synergistic functioning of the hybrid modifiers. These results underscore that the role of RPP transitions from a synergistic toughening agent in dense, reactive systems (e.g., SF) to a durability-oriented modifier that may compromise baseline strength in more porous systems (e.g., FA), highlighting the need for a systems-based design approach for cryogenic concrete [31].

### 3.2. Freeze–Thaw Resistance

**Figure 4:**
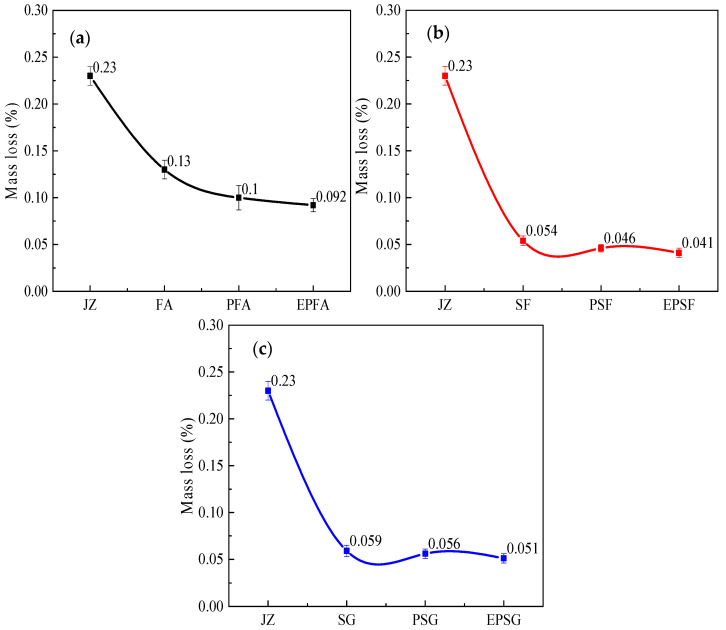
Mass loss rate of concrete with latex powder/PP fiber-modified mineral admixtures after freeze–thaw cycles at 20~−70 °C (**a**) FA groups, (**b**) SF groups and (**c**) SG groups.

**Figure 5:**
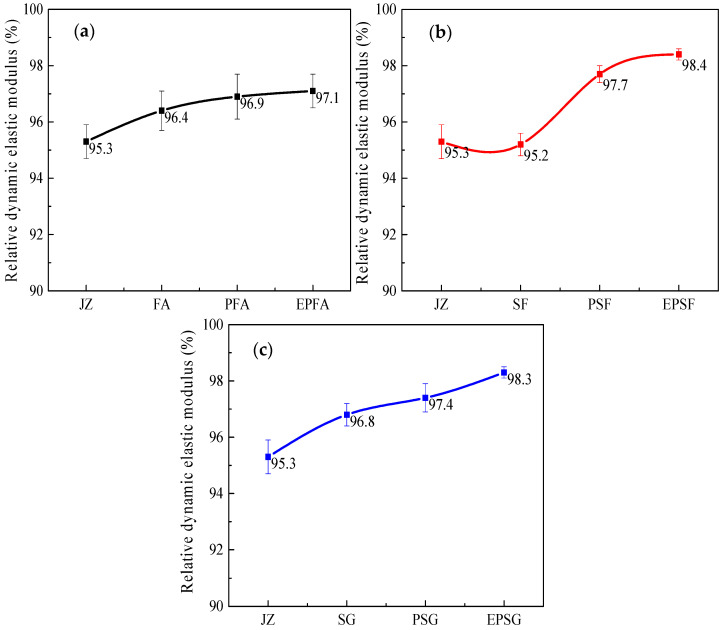
Relative dynamic elastic modulus of concrete with latex powder /PP fiber-modified mineral admixture after freeze–thaw cycles at 20~−70 °C (**a**) FA groups, (**b**) SF groups and (**c**) SG groups.

Frost resistance of concrete was assessed by mass loss and relative dynamic elastic modulus (RDEM) after 50 freeze–thaw cycles between +20 °C and −70 °C (Figure 4 and Figure 5). In the fly ash (FA) system, PP fibers and RPP polymer most effectively reduced mass loss after ULT cycling: the PFA mixture exhibited a 23.5% lower mass loss than the plain FA concrete. For the SF and SG systems, both fibers and hybrid modification reduced mass loss, albeit less effectively.

The relative dynamic elastic modulus (RDEM)—a key indicator of internal microdamage—exhibits a distinct hierarchy of improvement, underscoring a shift in the dominant protective mechanism depending on the mineral admixture (Figure 5). In the fly ash (FA) system, modifications provide only marginal benefits (PFA: +1.67% vs. JZ; EPFA: +0.21% vs. PFA), indicating that neither fiber bridging nor polymer film formation can significantly compensate for the system’s inherent porosity [32]. Conversely, the silica fume (SF) system exhibits a pronounced, fiber-dominated response. The PSF mixture shows the highest RDEM improvement (+2.5%), attributable to effective crack bridging by PP fibers within its dense microstructure. The additional minor gain from RPP indicates a secondary, complementary role for the polymer film. Most notably, the slag (SG) system reveals a clear polymer-dominated mechanism. Here, the hybrid EPSG mixture achieves the greatest overall enhancement (+3.1%), significantly outperforming the fiber-only PSG mix (+0.92%). This trend reversal highlights that, in the SG matrix, the flexibility, pore sealing, and enhanced cohesion provided by the RPP-derived polymer film become the primary factors in preserving structural integrity and damping freeze–thaw-induced vibrations, overshadowing the contribution of fiber reinforcement.

The observed freeze–thaw resistance trends align with those reported in previous studies of cementitious materials under cryogenic conditions. The polymer-dominated protection mechanism identified in the SG system—where the EPSG mixture showed the greatest overall improvement in relative dynamic elastic modulus (RDEM)—is consistent with Chen et al. [21], who also reported that polymer films enhance matrix cohesion and reduce permeability under cyclic thermal loading. Unlike their study, which focused on magnesium potassium phosphate cement, the present work demonstrates this mechanism in slag-based ordinary Portland cement. The marginal improvement observed in the FA system agrees with Rostasy et al. [9], who noted that more porous matrices are inherently more susceptible to ice crystallization pressure. Our SEM results support this finding: the FA system exhibited a 77% increase in void size after cycling, which explains its limited response to modification. Collectively, these comparisons confirm that the effectiveness of hybrid modification is intrinsically linked to the characteristics of the base matrix—a key finding demonstrated throughout this study.

### 3.3. Carbonation Resistance

Carbonation depth of concrete was measured to assess long-term durability under coupled ultra-low-temperature freeze–thaw cycling and natural carbonation (Table 4). The results reveal that both PP fibers and RPP reduce carbonation depth under standard conditions, with the hybrid-modified silica fume (SF) system (EPSF) exhibiting the best intrinsic resistance (0.67 mm at 28 d). However, after ULT freeze–thaw cycles, a clear divergence emerges between modification strategies. Mixtures containing only PP fibers (e.g., PFA, PSF) exhibit a marked increase in carbonation depth (0.14–0.17 mm), indicating that cyclic thermal stress weakens fiber–matrix interfaces and creates pathways for CO_2_ ingress [33]. In contrast, hybrid systems incorporating both RPP and fibers (e.g., EPFA, EPSF) show substantially smaller increases in carbonation depth after cycling. Notably, the fly-ash-based hybrid mixture (EPFA) exhibits the best performance, with the smallest increase (+0.06 mm), indicating that the polymer film effectively seals microcracks and interfacial defects induced by freeze–thaw action. These findings demonstrate that, although PP fibers enhance the initial microstructure, their durability under coupled environmental attack is substantially improved by the addition of RPP, which mitigates freeze–thaw damage and preserves long-term carbonation resistance.

The carbonation resistance results from this study are consistent with literature values reported for similar material systems. The excellent carbonation retention of the EPFA mixture—which showed the smallest increase in carbonation depth after freeze–thaw cycling (+0.06 mm)—is consistent with the findings of Schulze and Killermann [32], who demonstrated that polymer films can effectively seal microcracks and interfacial defects induced by environmental attack. Conversely, the more pronounced increase in carbonation depth observed in fiber-only mixtures (e.g., PFA, PSF) is in agreement with Ba et al. [33], who attributed this phenomenon to weakened fiber–matrix interfaces under cyclic thermal stress. Collectively, these comparisons confirm the superior performance of hybrid-modified systems—particularly those containing redispersible polymer powder (RPP)—in maintaining long-term durability under coupled environmental attack.

### 3.4. Early Age Crack Resistance

**Figure 6:**
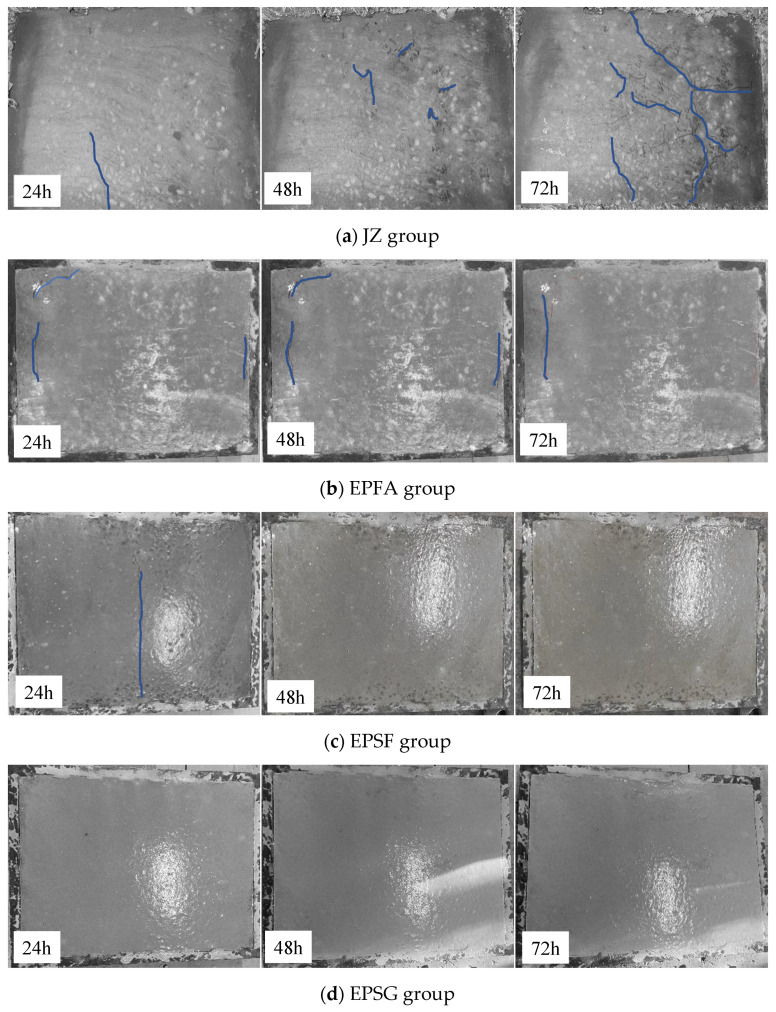
Crack patterns of concrete at 24 h, 48 h, and 72 h under restrained shrinkage conditions (Blue lines indicate the crack).

Early age cracking behavior, observed via restrained slab tests (Figure 6), reveals that modification with PP fibers and RPP substantially improves crack resistance, with efficacy strongly depending on the matrix. The plain reference concrete (JZ, Figure 6a) exhibits extensive and interconnected cracking. In contrast, all hybrid-modified systems show marked improvement. The most significant result is observed in the slag (SG)-based system (EPSG, Figure 6d), which displays no visible cracks throughout the 72 h test, corresponding to a perfect crack reduction coefficient (η) of 1.0 (Table 5).

The silica fume (SF) system (EPSF, Figure 6c) also performs well, with total crack area reduced to 6.85 mm^2^/m^2^ (η = 0.93). Although a single through-crack (44 mm long) develops at 24 h, it does not propagate further at 48 h and 72 h. The fly ash (FA) system (EPFA, Figure 6b) shows substantial improvement over the reference (η = 0.85), with total crack area reduced to 16.57 mm^2^/m^2^, though short, noncontinuous cracks appear primarily at the slab edges.

**Figure 7:**
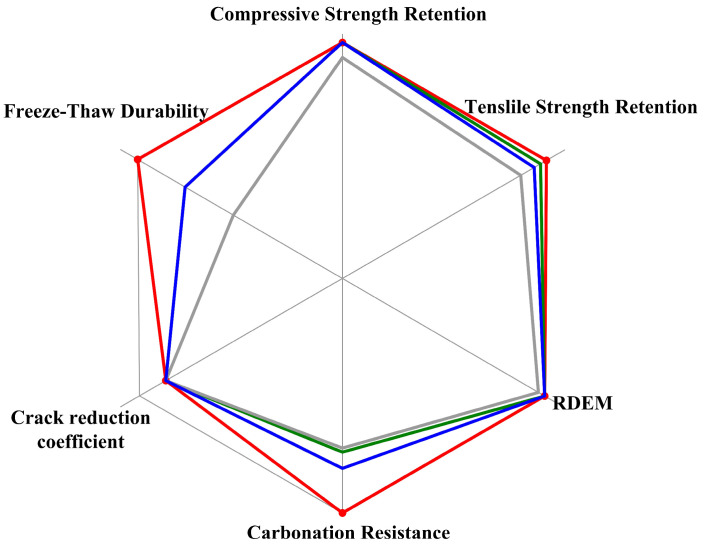
Performance Radar Chart Comparison (Post-ULT Cycling).

Figure 7 presents a radar chart comparing the post-ULT performance of four representative mixtures across six normalized indicators. The EPSF (silica fume with RPP and PP fibers) exhibits the largest and most symmetric polygonal area, achieving the highest or near-highest scores in all dimensions—particularly in strength retention, carbonation resistance, and frost mass retention. In contrast, EPFA (fly ash) shows a visibly smaller polygon with a distinct trade-off between durability and mechanical retention, while EPSG (slag) excels in RDEM and crack resistance but lags in carbonation resistance. The JZ reference occupies the smallest area, confirming the necessity of hybrid modification. This visualization quantitatively demonstrates that optimal cryogenic performance is not additive but requires matrix-enabled synergy, which is uniquely achieved in the silica fume system.

### 3.5. Microstructure Analysis

**Figure 8:**
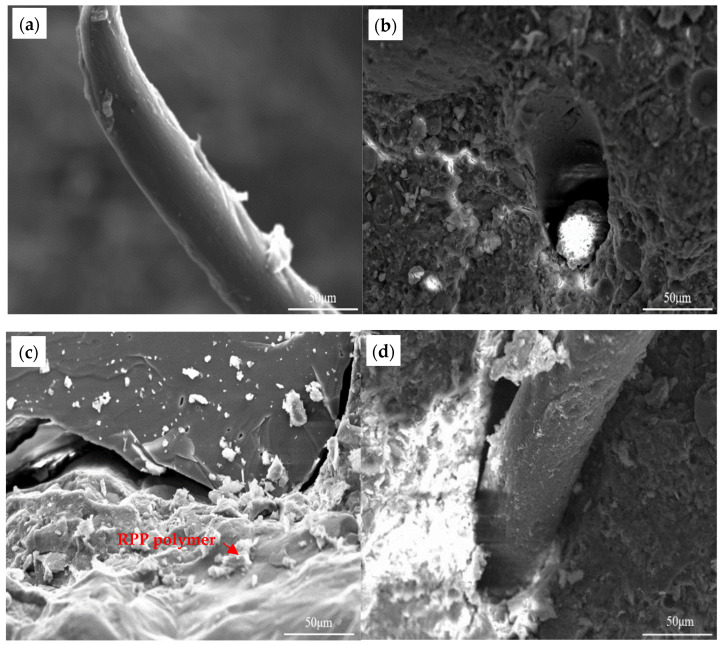
Microstructural evolution of the hybrid-modified FA system: (**a**) PP fiber-reinforced matrix before ULT cycling, showing fiber-matrix bonding; (**b**) the same after 50 ULT cycles; (**c**) RPP and PP fiber co-modified matrix before cycling, showing polymer phase distribution; (**d**) the same after ULT cycling, highlighting microstructural changes at interfaces.

**Figure 9:**
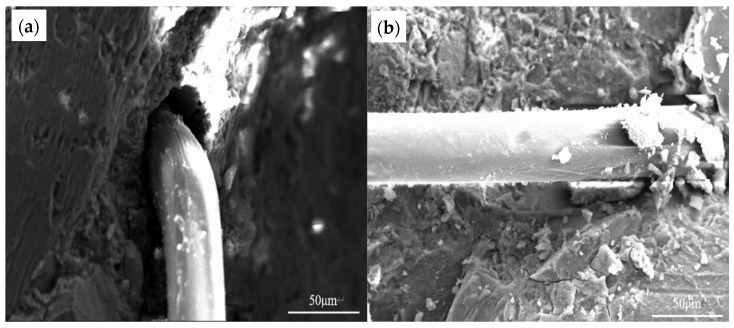

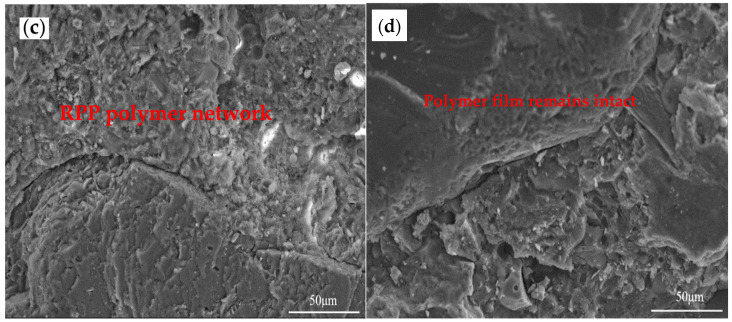
Microstructural evolution of the hybrid-modified SF system: (**a**) PP fiber-reinforced matrix before ULT cycling, showing dense hydration products; (**b**) the same after 50 cycles, maintaining matrix integrity; (**c**) RPP and PP fiber co-modified matrix before cycling, exhibiting seamless polymer-matrix integration; (**d**) the same after ULT cycling, demonstrating preserved microstructural cohesion and minimal damage.

**Figure 10:**
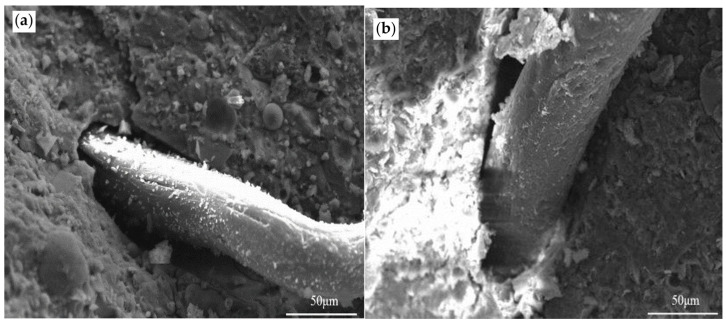

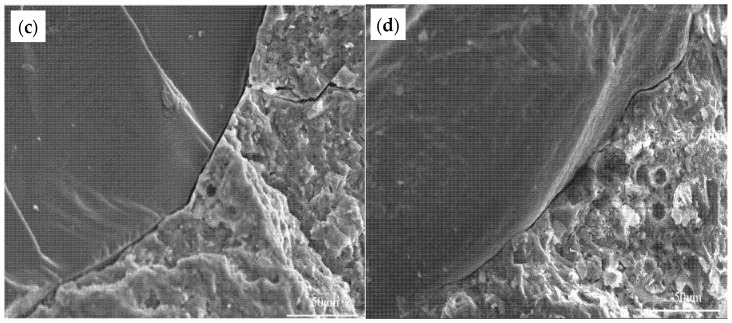
Microstructural evolution of the hybrid-modified SG system: (**a**) PP fiber-reinforced matrix before ULT cycling, showing typical fiber-matrix bonding; (**b**) the same after 50 ULT cycles, showing interfacial hydration products; (**c**) RPP and PP fiber co-modified matrix before cycling, illustrating uniform polymer distribution; (**d**) the same after ULT cycling, revealing coexisting secondary hydration features (spherical pits) and freeze–thaw damage patterns (honeycomb structure).

Microstructural analysis reveals fundamental mechanisms underlying the performance differences among mineral admixture systems. In all formulations, PP fibers show effective integration with the cementitious matrix, as evidenced by their serrated surfaces covered with hydration products (Figure 8a, Figure 9a and Figure 10a). The characteristic fracture morphology of these fibers—displaying tensile failure rather than interfacial debonding—provides direct evidence of their full mechanical engagement [34]. The fiber–matrix interaction provides the microstructural basis for the consistent enhancement in tensile properties, as the well-anchored fibers create a three-dimensional network that effectively bridges microcracks and redistributes stress [35].

The distribution and role of the polymer phase differ markedly among systems, explaining their divergent macroscopic responses. In the silica fume system (Figure 9c), RPP forms a continuous, interpenetrating network that integrates seamlessly with the dense SF matrix. This yields a homogeneous microstructure with few interfacial defects, which remains intact after ULT cycling (Figure 9d). This structural integrity underpins the superior durability and minimal strength loss of the EPSF mixture, as the cohesive matrix offers few pathways for damage propagation.

In contrast, the fly ash system exhibits a distinct polymer distribution pattern. As shown in Figure 8c, the polymer phase is localized at interfacial regions rather than being uniformly distributed. This spatial distribution correlates with the initial reduction in compressive strength evident in EPFA mixtures. However, the post-cycling microstructure (Figure 8d) reveals continued hydration and partial homogenization, indicating that ULT exposure still promotes microstructural evolution that partially offsets initial weaknesses.

The slag system exhibits competing processes under ULT conditions. Figure 10d reveals spherical features indicative of ongoing secondary hydration and localized regions with honeycomb structures characteristic of freeze–thaw damage. This coexistence explains the intermediate performance of SG-based mixtures, where property gains are partially offset by microstructural damage.

These observations demonstrate that achieving optimal performance under cryogenic conditions requires more than additive modifications—it demands microstructural compatibility. The superior performance of the SF system arises from the seamless integration of all components across multiple scales, creating a hierarchically reinforced composite. In other systems, the modifiers must overcome inherent matrix deficiencies, resulting in compensatory rather than synergistic behavior. This understanding provides critical guidance for designing advanced cryogenic concrete, highlighting that microstructural architecture ultimately determines performance.

To provide quantitative support for the microstructural observations, image analysis was performed on the SEM micrographs using ImageJ software (1.53f). The EPSF exhibited approximately 85% continuous polymer coverage throughout the matrix. It also maintained the smallest increase in average interfacial void size after ULT cycling (from 2.1 μm to 2.8 μm, a 33% increase). These microstructural features correlate with its lowest strength loss (6.7%) and most balanced durability performance. In contrast, the EPFA system showed only approximately 40% polymer coverage, localized at the fly ash particle interfaces, and a substantial increase in void size (from 3.5 μm to 6.2 μm, a 77% increase). This microstructural outcome is consistent with its highest strength loss (19.4%), while the localized interfacial sealing by the polymer also explains its best carbonation resistance. The EPSG system exhibited intermediate values for both polymer coverage (approx. 60%) and void size increase (from 2.8 μm to 4.5 μm, a 61% increase). This intermediate microstructural state reflects its intermediate strength retention and confirms its polymer-dominated protection mode, which is further evidenced by the greatest improvement in its dynamic elastic modulus. Collectively, these quantitative results provide direct support for the proposed mechanisms and confirm the consistency between the observed microstructural characteristics and the macroscopic performance of the mixtures.

### 3.6. Hydration Analysis

**Figure 11:**
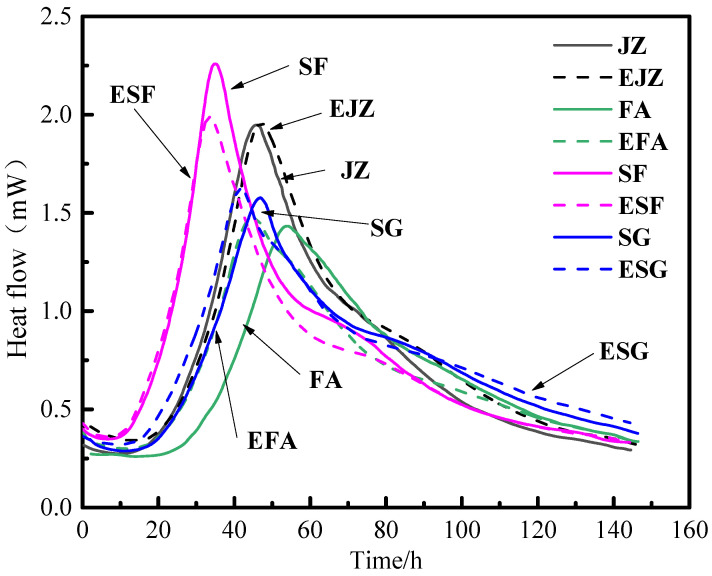
Effect of RPP on the hydration kinetics of mineral-admixtured cementitious systems.

Isothermal calorimetry reveals that redispersible polymer powder (RPP) does not act as a universal inhibitor or accelerator of hydration. Instead, it functions as a system-specific modulator, with its influence governed by the chemical and physical nature of the mineral admixture (Figure 11). This selective interaction fundamentally alters early age microstructure development, which in turn determines later-age durability under ultra-low-temperature (ULT) conditions.

For plain cement, RPP acts as a mild retarder. It increases the heat release rate during the induction period but delays the main hydration peak without affecting its peak intensity. This indicates that polymer particles initially provide nucleation sites but later form a transient film that moderately impedes ion diffusion, thereby extending the acceleration period.

In contrast, RPP functions as an activator in the slag (SG) system. It increases the heat release rate during both induction and acceleration periods, resulting in an earlier and more intense main hydration peak. This indicates that, in the SG system, the polymer enhances slag particle dissolution and facilitates hydration product precipitation, effectively catalyzing early hydration reactions. The silica fume (SF) system exhibits kinetic moderation. While RPP slightly advances the main peak, it significantly reduces its intensity and, notably, suppresses the heat release rate during the deceleration period. The overall effect is a flattened, prolonged heat evolution profile. This moderation is beneficial, as it reduces the internal temperature gradient and associated thermal stresses during early hydration. This contributes to the superior early age crack resistance observed in the SF-based hybrid mixtures [36].

The fly ash (FA) system exhibits an intermediate, complex response. RPP increases the early hydration rate, advancing the main peak, but simultaneously suppresses later-age heat release. This bifurcated effect—acceleration followed by suppression—indicates that the polymer initially enhances FA particle dispersion, thereby promoting early reactions, while its film-forming property later dominates and hinders long-term pozzolanic reaction kinetics [37]. This dual role corresponds to the macroscopic performance trade-off evident in the FA system: early age gains in workability and crack control are offset by compromised later-age strength development.

The hydration kinetics observed in each system are directly related to their subsequent performance under ULT conditions. In the EPSF, the flattened and prolonged heat evolution profile reduces early age thermal stress. This contributes to its excellent crack resistance (η = 0.93) and promotes the development of a dense microstructure, which underpins its lowest strength loss (6.7%) after ULT cycling. In the EPFA system, the initial acceleration followed by later-age suppression of hydration correlates with a strength–durability trade-off. Specifically, early polymer dispersion aids crack control, while the hindered pozzolanic reaction limits strength development. This combination explains its highest strength loss (19.4%) despite its excellent carbonation resistance. In the EPSG system, the enhanced hydration intensity reflects a polymer-activated slag reaction and secondary C-S-H formation. This leads to matrix densification, which in turn supports its greatest improvement in dynamic elastic modulus after ULT cycling. Collectively, these linkages demonstrate that early age hydration behavior directly influences the microstructural evolution that governs long-term cryogenic performance.

### 3.7. Nano-Mechanical Properties

To elucidate the microstructural origins of the divergent macroscopic performance of hybrid-modified concrete subjected to ultra-low-temperature (ULT) cycling, nanoindentation mapping was performed on selected specimens. Based on established ranges, C–S–H is classified into low-density (LD C–S–H, E ≈ 10–25 GPa), high-density (HD C–S–H, E ≈ 25–40 GPa), and ultra-high-density (UHD C–S–H, E ≈ 40–60 GPa). Unhydrated clinker exhibits a modulus >60 GPa [38,39]. The spatial distribution and relative abundance of these phases are significantly influenced by the mineral admixture and the presence of redispersible polymer powder (RPP).

**Figure 12:**
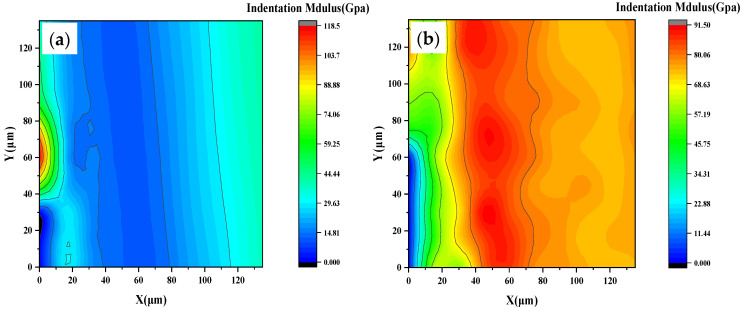
Elastic modulus distribution of tested points for samples prepared (**a**) JZ (**b**) EPSF.

Nanoindentation results reveal a clear contrast between the two systems (Figure 12). The plain cement matrix (JZ) exhibits a wide scatter in modulus values, ranging from approximately 20 to 120 GPa. This heterogeneity indicates a microstructure consisting largely of low-stiffness phases, such as LD C–S–H and porous regions, along with harder unhydrated particles. In contrast, the hybrid-modified silica fume system (EPSF) exhibits a notably uniform and higher modulus distribution, with values predominantly concentrated between 68 and 91 GPa. This marked microstructural refinement in the EPSF arises from a synergistic mechanism. Silica fume provides a highly reactive pozzolan that refines the pore structure and promotes the formation of dense C–S–H. The redispersible polymer powder (RPP) complements this by forming a cohesive film that enhances internal moisture retention—effectively supporting ongoing hydration—and improves the matrix’s stress redistribution capacity. The result is a homogeneous, high-stiffness microstructure with few weak interfaces.

The distinct modulus distributions observed provide direct micro-mechanical evidence for the divergent macroscopic performance of the mixtures. The narrow, high-modulus distribution observed in the EPSF (68–91 GPa) reflects a homogeneous microstructure dominated by dense C-S-H phases. Such a microstructure enables uniform stress distribution and limits damage initiation, a characteristic that directly contributes to its lowest strength loss (6.7%) after ULT cycling. In contrast, the wide modulus scatter in the JZ system (20–120 GPa) indicates a heterogeneous microstructure containing weak zones. These zones serve as preferential sites for microcrack formation, which explains its higher strength loss (12.8%) and greater susceptibility to freeze–thaw damage. This microstructural-mechanical relationship confirms that the homogeneous, high-modulus microstructure achieved in the EPSF provides the fundamental basis for its superior performance under ULT conditions.

## 4. Mechanistic Interpretation

**Figure 13:**
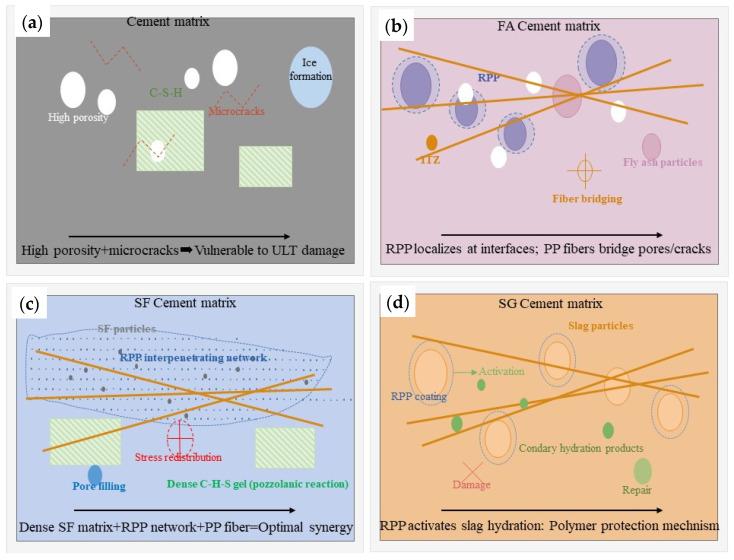
Microstructure and Synergistic Mechanisms of Hybrid-Modified Concrete Systems under Ultra-Low Temperature Conditions (**a**) JZ, (**b**) EPFA, (**c**) EPSF, (**d**) EPSG. (The orgin lines indicate the PP fibers).

The divergent performance of concrete under ULT cycling is governed by a fundamental principle: the interaction between redispersible polymer powder (RPP) and the cementitious matrix is dictated by selective compatibility, which is inherently determined by the mineral admixture type (Figure 13). This selectivity determines whether RPP and PP fibers yield synergistic enhancement or merely compensatory effects.

In the control system (JZ, Figure 13a), the unmodified porous matrix is susceptible to ice pressure and thermal stress damage [40]. The introduction of hybrid modifiers reveals distinct pathways of interaction. In the fly ash system (EPFA, Figure 13b), RPP localizes preferentially at FA particle boundaries. This mechanism seals the critical interfacial transition zones, resulting in the best post-freeze–thaw carbonation resistance. However, it simultaneously creates microregions with compromised mechanical continuity, leading to the observed strength–durability trade-off.

In contrast, the silica fume system (EPSF, Figure 13c) achieves optimal synergy. The pozzolanic reaction and nanofilling effect of SF create a dense, low-porosity microstructure [41]. Within this refined matrix, RPP forms a continuous, interpenetrating polymer network that cohesively binds hydration products, seals nanopores, and redistributes stress. This microscale network integrates seamlessly with the macroscale crack bridging provided by PP fibers, establishing a hierarchical, multiscale defense system that delivers balanced performance across all metrics.

The slag system (EPSG, Figure 13d) exhibits a polymer-activated competition mechanism. Here, RPP acts as a surface coating that enhances dissolution and secondary hydration of slag particles, promoting microstructural self-repair. This beneficial process competes with physical damage from ULT cycling, leading to a polymer-dominated protection mode that exhibits excellent crack resistance but intermediate frost durability.

Therefore, the role of RPP is not fixed but matrix-dependent: it acts as an interfacial sealer in FA, a cohesive network former in SF, and a hydration activator in SG. This work confirms that achieving true synergistic enhancement under ULT conditions requires more than simply adding modifiers. It necessitates a dense host matrix (e.g., with SF) that enables a cohesive polymer network, thereby unlocking the full potential of hierarchical reinforcement. This mechanistic understanding provides a foundational principle for the rational design of advanced cryogenic concrete.

## 5. Conclusions

This study investigated the synergistic effects of redispersible polymer powder (RPP) and polypropylene (PP) fibers on the ULT performance of concrete with different mineral admixtures (FA, SF, SG). The main findings and conclusions are summarized as follows:

(1) The hybrid modification exhibits selective compatibility. The performance enhancement from RPP and PP fibers is not universal but strongly depends on the physicochemical nature of the mineral admixture. This selectivity determines whether the modifiers act synergistically or compensatorily.
(2) The silica fume (EPSF) system exhibits optimal hierarchical synergy. The nanofilling and pozzolanic effects of SF create a dense microstructure, enabling RPP to form a continuous, interpenetrating polymer network. Coupled with macroscale PP fiber bridging, this multiscale integration yields balanced and superior ULT performance, including the highest residual strength (57.5 MPa), the lowest strength loss (6.7%), and excellent durability.
(3) Distinct mechanisms prevail in the FA and SG systems. In the FA system, RPP preferentially localizes at particle interfaces. This enhances durability—reflected in the best post-cycling carbonation resistance—but compromises initial monolithic strength, presenting a clear trade-off. In the SG system, RPP functions primarily as a hydration activator, promoting secondary C–S–H formation. This leads to polymer-dominated protection, as evidenced by the greatest improvement in dynamic elastic modulus and perfect early age crack resistance.
(4) A matrix-density-dependent synergy model is proposed. The results indicate that the effectiveness of RPP depends on the porosity and reactivity of the host matrix. Dense, fine-pored matrices (e.g., SF) enable uniform RPP distribution for bulk enhancement, whereas more porous matrices (e.g., FA) lead to localized RPP action for targeted interface repair.
(5) From a practical perspective, the silica fume-based hybrid system (EPSF) is recommended for LNG storage structures that require a balanced combination of mechanical and durability performance. In contrast, the slag-based system (EPSG) may be preferred in applications where early age crack control is of critical importance.

## Figures and Tables

**Table 1:** Chemical compositions of cementitious materials (wt.%).

Component	Cement	Fly Ash	Silica Fume	SG (Grade S95)
CaO	61.15	4.85	0.23	35.58
SiO_2_	22.36	54.76	96.82	36.10
Al_2_O_3_	7.21	24.56	0.21	16.32
Fe_2_O_3_	4.5	6.54		11.32
MgO	4.6			
TiO_2_		1.85		
LOI	0.18	7.44	2.74	0.68

**Table 2:** Parameters of redispersible latex powder.

Code	Composition	Non-Volatile Content (%)	Ash Content (%)	Bulk Density (g/L)	Mean Particle Size (μm)	PH
SWF-05	VAC/E	≥98.0	8–12	400–600	85 ± 15	5–8

**Table 3:** Mix proportions of concrete (kg/m^3^).

	Sand	Gravel	Cement	Water	SP	FA	SF	SG	PP	RPP
JZ	634.80	1179.02	434.3	152.18	5.07	0	0	0	0	0
FA	634.80	1179.02	347.11	152.18	5.07	86.96	0	0	0	0
SF	634.80	1179.02	407.98	152.18	5.07	0	26.09	0	0	0
SG	634.80	1179.02	347.11	152.18	5.07	0	0	86.96	0	0
PFA	634.80	1179.02	347.11	152.18	5.07	86.96	0.00	0.00	2.90	0.00
EPFA	634.80	1179.02	347.11	152.18	5.07	86.96	0.00	0.00	2.90	26.09
PSF	634.80	1179.02	407.98	152.18	5.07	0.00	26.09	0.00	2.90	0.00
EPSF	634.80	1179.02	407.98	152.18	5.07	0.00	26.09	0.00	2.90	26.09
PSG	634.80	1179.02	347.11	152.18	5.07	0.00	0.00	86.96	2.90	0.00
EPSG	634.80	1179.02	347.11	152.18	5.07	0.00	0.00	86.96	2.90	26.09

**Table 4:** Carbonation depth of concrete before and after ULT freeze–thaw cycling.

Mix ID	Carbonation Depth, 28d (mm)	Carbonation Depth, 150d (mm)	Depth After 50 FTCs, 28d (mm)	Δafter FTCs (mm)
JZ	0.92	1.95	1.36	+0.44
FA	1.15	2.41	1.38	+0.23
SF	0.90	1.88	1.05	+0.15
SG	1.08	2.22	1.25	+0.17
PFA	0.92	1.82	1.06	+0.14
EPFA	0.83	1.65	0.89	+0.06
PSF	0.82	1.70	0.96	+0.14
EPSF	0.67	1.42	0.83	+0.16
PSG	1.01	1.98	1.09	+0.08
EPSG	0.90	1.78	1.03	+0.13

**Table 5:** Evaluation of early cracking at 72 h.

Mix ID	Average Crack Area (mm^2^/Crack)	Crack Number (No./m^2^)	Total Crack Area (mm^2^/m^2^)	Crack Reduction Coefficient (η)
JZ	10.28	10.42	107.12	1 (reference)
EPFA	1.59	10.42	16.57	0.85
EPSF	3.3	2.08	6.85	0.93
EPSG	0	0	0	1

Note: η = A_J_/A_r,_ where A_J_ is the total crack area of reference mix JZ, and A_r_ is that of the tested mix.

## Data Availability

The original contributions presented in this study are included in the article. Further inquiries can be directed to the corresponding author.
